# Preclinical Histologic and Morphometric Comparison of Poly-L-Lactic Acid Fillers Formulated With Carboxymethyl Cellulose or Non-crosslinked Hyaluronic Acid in a Porcine Model

**DOI:** 10.7759/cureus.111360

**Published:** 2026-06-23

**Authors:** George Sulamanidze, Albina Kajaia, Dmitriy Nikishin

**Affiliations:** 1 Department of Plastic Surgery, Clinic “Total Charm Orbeliani”, Tbilisi, GEO; 2 Department of Cosmetic Dermatology, Total Charm Vake, Tbilisi, GEO; 3 Department of Research and Development, Aptos LLC, Tbilisi, GEO

**Keywords:** carboxymethyl cellulose, filler injection, hyaluronic acid, poly-l-lactic acid, tissue filler injections

## Abstract

Background: Poly-L-lactic acid (PLLA)-based injectable fillers are used as biostimulatory materials in aesthetic practice. Carrier components may influence particle distribution, hydration behavior, and tissue integration. Comparative preclinical data on PLLA formulations incorporating carboxymethyl cellulose (CMC) versus non-crosslinked hyaluronic acid (HA) remain limited.

Objective: To compare the longitudinal histologic and morphometric tissue response to PLLA fillers containing CMC or non-crosslinked HA in a porcine model.

Methods: In this paired preclinical study, 10 hybrid female pigs received bilateral subcutaneous injections of PLLA + CMC (Ellure CMC) and PLLA + HA (Ellure HA) at matched abdominal sites. Animals were euthanized at 7, 14, 30, 90, and 180 days post-injection (two animals per time point). Full-thickness skin and subcutaneous adipose tissue (SAT) were harvested from the injection sites. Histologic evaluation was performed using hematoxylin and eosin, Weigert-Van Gieson, and Sirius Red staining. Morphometric assessment included dermal thickness, fibroblast/fibrocyte-associated cellularity, vascular parameters, and collagen type I, collagen type III, and elastin content.

Results: Across all time points, neither formulation was associated with macroscopic inflammation or histopathologic evidence of overt adverse tissue reaction. Both formulations showed progressive tissue integration characterized by fibroblast/fibrocyte-associated cellular infiltration, neovascular remodeling, and changes in dermal thickness. Mean dermal thickness increased over time in both groups, from 928.6 ± 196.0 µm to 1590.1 ± 125.1 µm for Ellure CMC and from 1231.5 ± 267.8 µm to 1993.4 ± 149.5 µm for Ellure HA between days 7 and 180, with intermediate fluctuation. Collagen and elastin profiles showed broadly similar temporal patterns in skin and SAT in the two groups. Given the limited sample size and the exploratory design, these comparisons should be interpreted descriptively.

Conclusion: In this porcine model, PLLA fillers incorporating either CMC or non-crosslinked HA showed comparable descriptive histologic biocompatibility and tissue-remodeling profiles over 180 days. The findings support further controlled studies to determine whether carrier composition influences clinically relevant performance in cosmetic applications.

## Introduction

Poly-L-lactic acid (PLLA) and hyaluronic acid (HA) are widely used biocompatible polymers in aesthetic medicine, offering complementary mechanisms of action for skin rejuvenation. PLLA is a biodegradable synthetic polymer known for its biostimulatory effects, inducing a subclinical inflammatory response that promotes fibroblast activation and neocollagenesis, particularly of collagen types I and III. This process contributes to gradual volumization and dermal remodeling over several months [1-3]. HA, a naturally occurring glycosaminoglycan found abundantly in the extracellular matrix (ECM), enhances skin hydration, elasticity, and repair. In its non-crosslinked form, HA integrates seamlessly into dermal tissues, offering subtle volumization and hydration while also supporting fibroblast function [4-6].

In recent years, there has been growing interest in hybrid dermal filler formulations that combine PLLA with hydrating excipients such as HA or carboxymethyl cellulose (CMC). These dual-action formulations aim to merge the long-term collagen stimulation of PLLA with the immediate hydrating and rheological benefits of carrier molecules [7-9]. CMC is a water-soluble, plant-derived polysaccharide frequently used in pharmaceutical and cosmetic products for its viscosity-modifying, emulsifying, and stabilizing properties [10]. In injectable formulations, CMC serves not only as a suspending agent for microparticles but may also enhance the local tissue environment by modulating hydration and mechanical stability [11]. Unlike HA, which directly interacts with cellular receptors such as CD44, CMC exerts its effects primarily through its physicochemical properties; however, its contribution to the distribution and controlled biodegradation of PLLA particles may indirectly influence tissue remodeling [12,13].

Ellure (Aptos LLC, Tbilisi, Georgia) is a PLLA-based filler available in two distinct compositions: PLLA combined with CMC, and PLLA combined with HA. The carrier composition may influence the rheological properties, tissue integration, hydration capacity, and immediate volumizing effect of PLLA-based fillers. Formulations containing HA may provide greater initial hydration and immediate soft-tissue augmentation, potentially making them advantageous for superficial or dynamic facial areas where enhanced tissue integration and skin quality improvement are desired. In contrast, formulations containing CMC primarily function as suspension agents and may provide different handling characteristics and volumetric profiles. Nevertheless, both formulations primarily rely on PLLA-induced neocollagenesis for their long-term biostimulatory effect, and comparative clinical studies evaluating anatomical indication-specific superiority remain limited. Ellure with CMC contains 80% pure PLLA (160 mg) and 20% CMC (40 mg). CMC is a water-soluble polysaccharide derived from cellulose and is valued for its biocompatibility and ability to stabilize PLLA microparticles [14-16]. Its plant-based origin also reduces immunogenic risk compared to animal-derived fillers, and the absence of cellulase in humans helps preserve its mechanical stability in vivo. Ellure with HA consists of 85% pure PLLA (170 mg) and 15% non-crosslinked HA (30 mg). The non-crosslinked HA is recognized for improving skin hydration and elasticity without producing the pronounced volumizing effects characteristic of crosslinked HA. Due to its smooth integration into the dermal matrix, this form of HA provides immediate hydration, promotes collagen synthesis, and maintains the natural contours of the face and body [17,18].

The PLLA in Ellure is characterized by defined microsphere morphology and particle-size distribution, features that may influence tissue dispersion and local integration after implantation. The reported particle size range of 20-30 µm may reduce particle aggregation and support more uniform distribution within the tissue. In addition, the presence of both smooth and porous microspheres may contribute to progressive biodegradation and sustained tissue remodeling. These formulation characteristics provide a rationale for comparative preclinical evaluation.

Among preclinical animal models, pigs (porcine models) are particularly advantageous because their skin is anatomically and physiologically similar to human skin, with comparable thickness, collagen composition, and hair follicle structure [14,15]. This close resemblance enables more accurate predictions of a filler’s behavior in human tissue. The aim of this study was to perform a long-term comparative assessment of skin histology and morphometric tissue responses following injection of PLLA-based fillers containing either CMC or non-crosslinked HA in a porcine model.

## Materials and methods

Study design and ethical approval

This exploratory preclinical study was conducted in accordance with the Animal Research: Reporting of In Vivo Experiments (ARRIVE) 2.0 guidelines for reporting animal research [16] and was approved by the Ethics Committee of the Preclinical Research Center, Penza, Russian Federation (Protocol No. 5-2023; August 23, 2023). The study was designed to compare the local tissue response to two PLLA-based filler formulations differing in carrier composition: one containing CMC and the other containing non-crosslinked HA.

A paired within-animal design was used in order to minimize inter-animal variability and allow direct descriptive comparison of local tissue responses under matched biological conditions.

Animal model

Ten first-generation hybrid female pigs (Mangalitsa × Duroc), aged five months and weighing approximately 50 kg each, were selected for the experiment. Animal demographics are summarized in Table 1. Inclusion criteria required animals to be in optimal health, as confirmed by comprehensive hematological and biochemical screening (hemoglobin ≥110 g/L).

**Table 1:** Demographic and baseline characteristics of experimental pigs.

Parameter	Description
Species	Domestic pig (*Sus scrofa domesticus*)
Breed	Hybrid (Mangalitsa × Duroc)
Generation	First-generation hybrid
Sex	Female
Number of animals	10 pigs
Age	Five months
Sexual maturity	Sexually mature
Body weight	55-60 kg
Genetic background	Genetically similar siblings from the same litter
Health status	Clinically healthy based on a comprehensive blood test
Hemoglobin inclusion criterion	≥110 g/L
Termination groups	7, 14, 30, 90, and 180 days

Exclusion criteria included evidence of systemic illness on clinical examination; abnormal hematological or biochemical screening results below the predefined inclusion thresholds (including hemoglobin <110 g/L); signs of infection or dermatologic disease at potential injection sites; pregnancy; failure to acclimatize or tolerate handling/anesthesia; and any peri-procedural complication that could confound local tissue response assessment. Animals meeting any exclusion criterion were not enrolled or were withdrawn prior to scheduled procedures.

All animals were housed under standardized conditions: temperature 21 ± 2°C, relative humidity 30-60%, and a 12-hour light/dark cycle. Animals were acclimatized for one week prior to the initiation of experimental procedures. Animals received a standardized controlled diet throughout the study period, formulated to maintain stable metabolic and general health conditions during follow-up.

Randomization and allocation of injection sites

**Figure 1:**
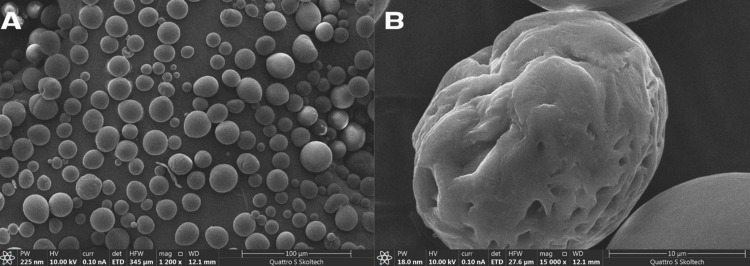
Microphotographs of PLLA particles. Scanning electron microscopy images obtained using a low-vacuum scanning electron microscope (LV-SEM; JSM-5910LV, JEOL Ltd., Tokyo, Japan). (A) Low-magnification image at 1,200× magnification. (B) High-magnification image at 15,000× showing detailed surface morphology of the particles.

A paired within-animal design was used. Each animal received both investigational formulations, injected bilaterally into comparable abdominal sites located approximately 2 cm lateral to the nipple line. Allocation of formulation to the left or right side was predetermined under standardized conditions to ensure consistent anatomical placement and facilitate subsequent tissue harvesting. Representative microphotographs of the PLLA particles used in the investigational formulations are shown in Figure 1.

Injection procedure

Each animal received both investigational formulations at matched bilateral abdominal sites. Injection zones were located approximately 2 cm lateral to the nipple line in order to ensure consistent anatomical placement and facilitate subsequent tissue harvesting. After reconstitution, 0.3 mL of each formulation was administered subcutaneously using a standardized bolus injection technique under sterile conditions. The selected injection volume was intended to provide sufficient localized material deposition for reliable histological and imaging evaluation while minimizing excessive tissue distension, leakage, or overlap between adjacent injection sites.

This implantation approach was selected to ensure precise localization of the injected material for subsequent histologic assessment. Although appropriate for controlled preclinical sampling, this method does not fully replicate routine clinical injection techniques used in aesthetic practice, where PLLA-based products are typically distributed using linear threading, fanning, cross-hatching, or multilayered injection patterns across broader anatomical areas. In contrast, bolus injection results in a more concentrated focal deposition of material, which may influence local tissue response, including inflammatory cell distribution, foreign body reaction intensity, particle aggregation, and collagen remodeling dynamics. Nevertheless, the standardized bolus approach enabled reproducible comparison between formulations under controlled experimental conditions.

Anesthesia and perioperative care

All procedures were performed under general anesthesia in sterile surgical conditions. Anesthesia was induced using intramuscular xylazine and Zoletil 100, followed by maintenance with inhalational isoflurane and intravenous propofol as required. Physiologic monitoring included heart rate, arterial pressure, and oxygen saturation throughout the procedure. Prophylactic ceftriaxone was administered perioperatively. Recovery from anesthesia occurred within approximately 30-40 minutes after completion of the procedure.

Tissue sampling and histological preparation

Animals were euthanized at 7, 14, 30, 90, and 180 days after injection, with two animals evaluated at each time point. Full-thickness tissue specimens, including skin and subcutaneous adipose tissue (SAT), were excised from each injection site. Five tissue fragments were obtained from each injection site for histologic processing. These represented technical replicate samples from the same implantation field rather than independent biological replicates.

Histological staining and imaging

Excised tissues were fixed in 7% neutral buffered formalin, processed routinely, embedded in paraffin, and sectioned at 5-7 µm. Hematoxylin and eosin staining was used to evaluate overall tissue architecture, cellularity, and the presence of inflammatory or foreign-body responses. Weigert-Van Gieson staining was performed to visualize connective tissue organization, including elastin fiber distribution within the dermis and stromal septa of the SAT. Sirius Red staining was used to assess collagen fiber content and distribution, with polarized light microscopy applied to support differentiation of collagen fiber characteristics.

Digital micrographs were acquired using brightfield and polarized light microscopy at 40×, 100×, and 200× magnification with a 12-megapixel Sony imaging sensor (Sony Corporation, Tokyo, Japan). Morphometric analysis was performed on harvested tissue sections and included measurement of dermal thickness, fibroblast/fibrocyte-associated cellularity, blood vessel count, and blood vessel diameter. In addition, the relative proportions of collagen type I, collagen type III, and elastin were assessed in both skin and SAT. These measurements were used to characterize temporal patterns of tissue integration and ECM remodeling following implantation.

Statistical analysis

Statistical analysis was performed using STATISTICA version 7.0 (StatSoft Inc., Tulsa, Oklahoma, USA). Descriptive data were summarized as mean ± standard error (SE), together with median, interquartile range (IQR), and observed minimum-maximum values where applicable.

Because only two animals were evaluated at each time point and multiple tissue fragments were sampled from the same local implantation field, the dataset should be interpreted primarily as descriptive and exploratory. Although the original analytical plan included nonparametric paired comparisons using the Wilcoxon signed-rank test, the study was not powered for robust inferential analysis across time points. Accordingly, the results are interpreted conservatively, with emphasis on descriptive longitudinal and between-formulation trends. Where exploratory hypothesis testing was performed, a two-sided p-value < 0.05 was considered statistically significant.

## Results

Macroscopic findings

No overt macroscopic signs of adverse local tissue reaction were observed at any injection site throughout the study period in either treatment group. Specifically, no visible erythema, edema, ulceration, necrosis, or gross inflammatory changes were identified following implantation of either formulation.

At the Ellure CMC injection sites, mild palpable induration was noted at the early observation points (days 7 and 14), became less apparent by day 30, and was no longer detectable at days 90 and 180. At the Ellure HA injection sites, no palpable induration was identified by day 14; however, mild late induration was noted at days 90 and 180 in the absence of associated gross inflammatory change. Overall, these findings did not indicate clinically evident adverse reactivity and were considered consistent with temporal variation in local tissue remodeling after implantation.

Microscopic findings

**Figure 2:**
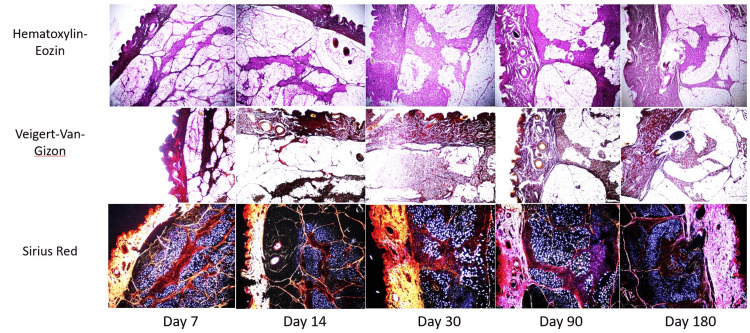
Microphotographs of samples containing skin and SAT of pigs at sites of injection of Ellure CMC filler, stained using hematoxylin-eosin, Weigert-Van Gieson, or Sirius Red method. Tissue samples were collected 7, 14, 30, 90, 180 days after filler injection. Magnification: x40. SAT: subcutaneous adipose tissue; CMC: carboxymethyl cellulose

**Figure 3:**
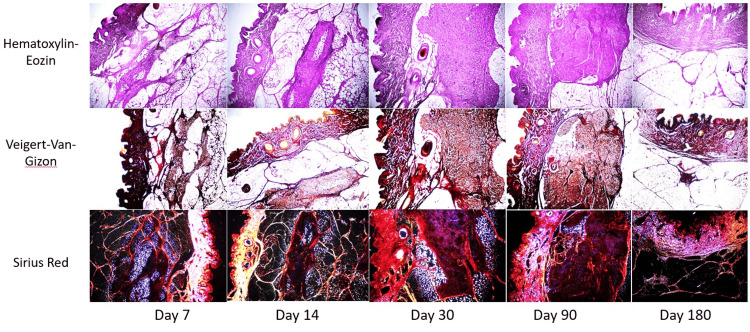
Microphotographs of samples containing skin and SAT of pigs at sites of injection of Ellure HA filler, stained using hematoxylineosin, Weigert-Van Gieson, or Sirius Red method. Tissue samples were collected 7, 14, 30, 90, 180 days after filler injection. Magnification: x40. SAT: subcutaneous adipose tissue; HA: hyaluronic acid

Histologic examination demonstrated preservation of the overall architecture of both skin and SAT at all time points in both groups. No destructive tissue changes, overt suppurative inflammation, necrosis, or pathologic foreign-body reaction were identified (Figures 2, 3). The physiological process of tissue regeneration, including neoangiogenesis, was evident following the intervention. The degree of hydration of dermis and subcutaneous tissue varied across the analyzed timepoints.

Post-implantation tissue responses

Histological examination of slides stained with hematoxylin and eosin revealed no pathological changes in the cellular composition of the skin and SAT. Qualitative tissue-response parameters, including the presence or absence of a well-defined fibrous capsule, fibroblast-associated cellularity, hydration, blood flow, and neoangiogenesis, are summarized in Table 2.

**Table 2:** Tissue responses to Ellure CMC and Ellure HA observed at 7, 14, 30, 90, and 180 days after filler injection. CMC: carboxymethyl cellulose; SAT: subcutaneous adipose tissue; HA: hyaluronic acid

Post-implantation day	Ellure CMC	Ellure HA
Day 7	Poorly defined capsule primarily composed of cellular elements	No well-defined capsule
	Moderate presence of fibroblasts	Small number of fibroblasts
	Moderate dermal hydration and mild SAT hydration near the implant site	Mild hydration in both the dermis and SAT around the implant
	Increased blood flow around the implant and initiation of neoangiogenesis	Increased blood flow around the implant and initiation of neoangiogenesis
Day 14	Poorly defined capsule composed primarily of cellular elements	No well-defined capsule
	Significant number of fibroblasts	Notable accumulation of fibroblasts surrounding the implant
	Moderate hydration in both the dermis and SAT near the implant site. Active neoangiogenesis, blood flow increased around the implants	Significant dermal hydration and moderate SAT hydration in the implantation area. Active neoangiogenesis, blood flow increased around the implants
Day 30	No well-defined capsule	No well-defined capsule
	Moderate number of fibroblasts	Significant presence of fibroblasts
	Mild dermal and SAT hydration	Moderate hydration in the dermis and mild hydration in the SAT near the implant site
	Elevated blood flow, active neoangiogenesis	Elevated blood flow, active neoangiogenesis
Day 90	No well-defined capsule	No well-defined capsule
	Moderate numbers of fibroblasts around the implants	Moderate numbers of fibroblasts around the implants
	Significant dermal hydration and mild SAT hydration near the implant site	Moderate dermal hydration and significant SAT hydration near the implantation area
	Less pronounced neoangiogenesis	Less pronounced neoangiogenesis
	Blood flow increased	Blood flow increased
Day 180	No well-defined capsule	No well-defined capsule
	Moderate numbers of fibroblasts around the implant	Moderate numbers of fibroblasts around the implant
	Mild dermal and SAT hydration near the implant site	Moderate dermal hydration and minimal SAT hydration in the implantation area
	Increased blood flow around the implant	Reduced blood flow around the implant

Dermal thickness

Throughout the study, descriptive analysis suggested a gradual overall increase in mean dermal thickness for both fillers (Table 3).

**Table 3:** Dermal thickness observed at 7, 14, 30, 90, 180 days after injection of Ellure CMC or Ellure HA. Data presented as mean ± SE for five skin samples. CMC: carboxymethyl cellulose; HA: hyaluronic acid; SE: standard error of the mean

Day after injection	Ellure CMC	Ellure HA
Dermal thickness (µm), mean ± SE		
7	928.6 ± 196.0	1231.5 ± 267.8
14	1204.1 ± 181.8	1319.42 ± 290.4
30	1552.4 ± 139.5	1899.44 ± 153.4
90	2114.8 ± 181.5	1778.93 ± 270.0
180	1590.1 ± 125.1	1993.4 ± 149.5

Skin and SAT morphology and composition

Tables 4, 5 present measurements of dermal and stromal-vascular components based on hematoxylin-eosin-stained tissue sections at different time points following implantation: fibrocyte and blood vessel count, diameter of blood vessels. Elastic fibers were visualized with Weigert-Van Gieson staining in the thickness of the dermis and in the connective tissue interlayers of the SAT for both filler materials at all assessed intervals.

**Table 4:** Results of measurements of dermal and stromal-vascular components in tissue samples of skin and SAT observed at 7, 14, 30, 90 and 180 days after injection of Ellure CMC filler. Data presented as mean ± SE for five skin samples. CMC: carboxymethyl cellulose; SAT: subcutaneous adipose tissue; SE: standard error of the mean

Days	7 days	14 days	30 days	90 days	180 days
Number of fibrocytes (units)					
Skin	3219.2 ± 956.5	5376.7 ± 1802.4	4452.1 ± 320.4	2020.6 ± 851.0	6746.6 ± 1017.4
SAT	2979.5 ± 817.6	6164.4 ± 1252.5	5411.0 ± 2099.3	3972.6 ± 2170.7	8698.6 ± 2500.2
Diameter of blood vessels (µm)					
Skin	23.0 ± 6.1	23.2 ± 5.0	37.5 ± 2.5	32.4 ± 14.8	35.0 ± 16.1
SAT	44.7 ± 30.5	55.7 ± 18.2	79.3 ± 14.7	35.5 ± 7.7	50.7 ± 41.3
Number of blood vessels per 0.7 mm^2^					
Skin	10.8 ± 2.4	7.4 ± 2.3	7.60 ± 2.19	4.80 ± 2.17	6.00 ± 1.22
SAT	5.2 ± 1.5	5.4 ± 1.3	7.0 ± 1.9	7.6 ± 2.6	3.8 ± 1.6

**Table 5:** Results of measurements of dermal and stromal-vascular components in tissue samples of skin and SAT observed at 7, 14, 30, 90 and 180 days after injection of Ellure HA filler. Data presented as mean ± SE for five skin samples. HA: hyaluronic acid; SAT: subcutaneous adipose tissue; SE: standard error of the mean

Days after injection	7 days	14 days	30 days	90 days	180 days
Number of fibrocytes (units)					
Skin	2294.5 ± 826.5	5102.7 ± 1231.2	4452.1 ± 605.4	2363.0 ± 1182.6	4006.9 ± 636.1
SAT	3938.4 ± 1062.5	7808.2 ± 2318.3	5000.0 ± 779.1	2534.3 ± 505.1	3664.4 ± 1038.8
Diameter of blood vessels (µm)					
Skin	31.0 ± 5.0	27.1 ± 8.2	43.0 ± 20.3	26.4 ± 5.2	36.7 ± 20.2
SAT	33.1 ± 5.2	47.5 ± 14.1	37.0 ± 18.5	69.7 ± 43.1	39.0 ± 25.2
Number of blood vessels per 0.7 mm^2^					
Skin	9.4 ± 2.3	8.0 ± 2.0	7.6 ± 1.8	5.8 ± 1.8	5.6 ± 2.0
SAT	7.4 ± 1.3	5.0 ± 1.0	7.8 ± 0.8	9.0 ± 0.7	4.4 ± 0.9

Throughout the observation period, fibrocyte count, blood vessel diameter, and blood vessel count in both the skin and SAT were similar in the Ellure CMC- and Ellure HA-treated skin samples, with a potential difference in fibrocyte count on day 180.

On day 7, blood vessel counts in the skin were relatively high while blood vessel diameters were relatively small (Tables 4, 5), indicating the presence of neoangiogenesis. Single collagen fibers were observed at short distances from the implant edge. On day 14 post-implantation, in the Ellure CMC samples, multiple collagen fibers were observed at a considerable distance from the implant’s edge. In tissues treated with Ellure HA, multiple collagen fibers were dispersed throughout the implant site. On days 30, 90, and 180, multiple collagen fibers were distributed through the entire depth and length of both Ellure CMC and Ellure HA implants.

Collagen and elastin fiber content

The percentages of collagen types I and III, as well as elastin in the skin and SAT, are presented in Table 6. Following an initial period of stability, a gradual modulation of collagen type I levels was observed in both the skin and SAT from day 90 onward, with a similar pattern noted for collagen type III in the SAT. Elastin levels in the skin remained consistently stable throughout the study period. Despite the inherent variability and limited sample size, the overall tissue response to both Ellure CMC and Ellure HA demonstrates a comparable and consistent profile. These findings indicate that both formulations elicit similar biological effects, supporting their potential as effective biostimulators.

**Table 6:** Percentages of collagen types I and III and elastin in tissue samples of skin and SAT observed at 7, 14, 30, 90 and 180 days after injection of Ellure CMC or Ellure HA filler. Data presented as mean ± SE for five skin samples. CMC: carboxymethyl cellulose; HA: hyaluronic acid; SAT: subcutaneous adipose tissue; SE: standard error of the mean

Parameter	Days after injection	7 days	14 days	30 days	90 days	180 days
% of total fibers in the skin						
Collagen type I	Ellure CMC	80.2 ± 9.8	82.9 ± 8.0	94.4 ± 2.7	71.6 ± 7.9	64.2 ± 2.5
	Ellure HA	86.1 ± 7.0	91.8 ± 4.7	90.0 ± 3.3	69.3 ± 12.2	58.3 ± 19.1
Collagen type III	Ellure CMC	4.6 ± 3.8	1.6 ± 0.8	0.9 ± 0.7	1.3 ± 0.3	2.3 ± 1.2
	Ellure HA	0.8 ± 0.3	1.2 ± 1.0	0.7 ± 0.2	2.1 ± 0.6	4.3 ± 3.7
Elastic fibers	Ellure CMC	1.2 ± 0.2	1.2 ± 0.4	2.4 ± 3.2	1.5 ± 0.7	1.4 ± 1.1
	Ellure HA	1.3 ± 0.1	1.2 ± 0.3	1.1 ± 0.2	1.2 ± 0.4	2.2 ± 1.8
% of total fibers in SAT						
Collagen type I	Ellure CMC	34.5 ± 7.1	28.5 ± 5.8	29.8 ± 11.8	20.9 ± 11.3	8.7 ± 2.8
	Ellure HA	41.0 ± 8.9	42.9 ± 6.0	60.5 ± 19.0	17.6 ± 7.7	7.2 ± 4.1
Collagen type III	Ellure CMC	6.5 ± 1.5	11.3 ± 2.4	3.8 ± 1.1	2.6 ± 0.4	2.0 ± 0.4
	Ellure HA	6.3 ± 1.6	13.2 ± 2.8	2.0 ± 0.6	1.5 ± 0.3	1.2 ± 0.9
Elastic fibers	Ellure CMC	0.5 ± 0.3	0.3 ± 0.4	5.2 ± 1.6	1.9 ± 1.0	4.0 ± 0.6
	Ellure HA	1.4 ± 0.6	0.6 ± 0.4	1.5 ± 1.4	0.3 ± 0.3	3.7 ± 0.6

Thus, the descriptive analysis of dermal and subcutaneous tissue morphology following the implantation of Ellure CMC and Ellure HA revealed comparable trends in fibrocyte presentation, blood vessel development, and ECM remodeling over the six-month study period. Both fillers demonstrated similar patterns of collagen and elastin deposition, suggesting consistent tissue integration without notable differences in structural composition.

## Discussion

This study aimed to evaluate and compare the long-term tissue responses to two PLLA-based dermal fillers - Ellure CMC and Ellure HA - in a porcine model. Both formulations demonstrated favorable histological profiles, including fibroblast activation, dermal thickening, neovascularization, and ECM remodeling, without evidence of mature fibrous capsule formation or significant inflammation. At early time points in the Ellure CMC group, a transient poorly organized cellular rim was observed; however, this did not persist and was not consistent with mature pathologic encapsulation. These findings align with the known mechanism of action of PLLA, which exerts a biostimulatory effect through low-grade inflammatory activation of macrophages and fibroblasts, leading to progressive neocollagenesis and tissue volumization [18,19].

Our results are consistent with previous preclinical studies. Cabral et al. showed that PLLA-based formulations increase collagen synthesis in vitro and in vivo, with progressive tissue integration over time [20]. Similarly, Herrmann et al. described the ability of PLLA microspheres to support long-term soft tissue augmentation without triggering adverse immune responses [21]. In the present study, both the CMC and non-crosslinked HA carriers appeared to facilitate uniform dispersion of PLLA microspheres and supported comparable tissue remodeling, suggesting that both excipients are suitable vehicles for sustained biostimulation.

Notable differences between the two formulations were observed in hydration dynamics and vascularization patterns. The Ellure HA group showed delayed but clinically insignificant induration at later time points, which may reflect prolonged hydration and vascular activity due to HA’s known bioactivity. HA can enhance endothelial proliferation and modulate local inflammatory signaling, primarily through its interactions with CD44 and RHAMM receptors [22-24].

The reduction in blood flow observed in the Ellure HA group at the 180-day time point may reflect the transition from an active remodeling phase toward a more mature and stabilized tissue response. Early increases in vascularization are commonly associated with inflammatory activation, fibroblast recruitment, neocollagenesis, and ECM remodeling induced by PLLA-based biomaterials. As tissue maturation progresses and the reparative process stabilizes, metabolic demand and angiogenic signaling may decline, resulting in reduced vascular density and blood flow. In addition, partial biodegradation of the HA carrier over time may alter local hydration and tissue microenvironment characteristics, potentially contributing to reduced vascular activity at later stages. Importantly, this finding was not associated with histological evidence of adverse tissue reaction or necrosis, suggesting that the decreased blood flow more likely reflects physiological tissue maturation rather than pathological ischemic change.

To our knowledge, this is one of the first studies directly comparing PLLA-based fillers formulated with CMC and non-crosslinked HA in a large animal model. Previous research has largely focused on PLLA alone or compared it to HA fillers with volumizing intent [25,26].

While the results are promising, several limitations must be acknowledged. The sample size was limited, with only two animals examined per time point, which restricts statistical power and precludes broad inferential conclusions. Additionally, multiple histological fragments were obtained from the same injection site, representing technical rather than biological replicates. The bolus injection technique used here differs from clinical practice, where fillers are typically administered in smaller aliquots over wider areas. Furthermore, only histological and morphometric parameters were evaluated; no molecular, mechanical, or functional assessments were performed. The absence of a PLLA-only control group also limits our ability to isolate the specific contributions of the CMC and HA carriers. Future studies incorporating larger cohorts, functional endpoints, and longer follow-up durations will be essential to build upon these findings.

Future clinical studies should further evaluate not only the histological behavior of PLLA-based hybrid fillers but also clinically relevant outcomes such as longevity of correction, tissue palpability, skin hydration, elasticity, patient-reported skin quality improvement, and performance across different anatomical regions. In particular, comparative assessment of dynamic facial areas, regions with thinner skin, and body applications may help clarify whether differences in carrier composition influence clinical integration, tissue behavior, or aesthetic outcomes over time. Longitudinal evaluation using imaging modalities, biomechanical skin measurements, and validated patient-reported outcome scales would further enhance understanding of the long-term performance and safety profile of these formulations.

## Conclusions

In this porcine model, both Ellure CMC and Ellure HA were associated with acceptable descriptive histologic biocompatibility and sustained tissue-remodeling patterns over a 180-day period. Despite differences in carrier composition, both formulations showed broadly comparable qualitative and morphometric responses within the limits of this exploratory preclinical design. These findings support further controlled and appropriately powered studies to determine whether carrier composition influences clinically relevant performance in cosmetic applications.
